# Otolaryngological Presentations of Klippel-Feil Syndrome: A Systematic Review

**DOI:** 10.7759/cureus.73986

**Published:** 2024-11-19

**Authors:** Christopher Stewart, Alex L Otto, Mitchell Fisher, Abbigail Niewchas, Salma Alkhatib, Andrew Simonsen, Randall Hansen, Suporn Sukpraprut-Braaten, Kent McIntire

**Affiliations:** 1 College of Medicine, Kansas City University, Joplin, USA; 2 Otolaryngology - Head and Neck Surgery, Freeman Health System, Joplin, USA; 3 Graduate Medical Education, Kansas City University, Joplin, USA

**Keywords:** hearing loss, klippel-feil syndrome, otolaryngology, rare genetic disorder, spinal cord fusion

## Abstract

Klippel-Feil syndrome (KFS) is a rare congenital condition characterized by the fusion of cervical vertebrae. It classically presents with a triad of symptoms: limited cervical range of motion, a low posterior hairline, and a short neck. Common otolaryngological manifestations include hearing loss, dysphagia, cleft palate, jaw disorders, thyroid abnormalities, and ear malformations, highlighting the importance of KFS awareness in the field of otolaryngology. Recognizing these symptoms can enhance patient care and outcomes. This systematic review analyzed all case reports on KFS published in the last 10 years on PubMed. Cases were classified using the Samartzis classification, Mallampati score, and Cormack-Lehane grade. Symptoms were then identified, and common imaging techniques were noted to provide clinical recommendations for treating KFS patients. The study found that more severe vertebral fusions are linked to more serious symptoms. Otolaryngologists should consider KFS in their differential diagnosis, especially in patients presenting with neck masses and pain, hearing loss, dysphagia, scoliosis, Sprengel's deformity, or other musculoskeletal abnormalities.

## Introduction and background

Klippel-Feil syndrome (KFS) is a rare congenital fusion of the cervical vertebrae [1]. Patients with KFS may present with a classic triad of limited cervical range of motion, a low posterior hairline, and a short neck [2]. It often occurs with deformities such as torticollis and Sprengel’s deformity [3,4]. The occurrence is rare, affecting approximately 1 in 40,000 live births [5,6]. Although sources indicate that occurrence may be much higher than this, it often goes undiagnosed [7]. While there have been several genes linked to KFS such as GDF6, GDF3, and MEOX1, the exact cause remains unknown and is often sporadic [7,8]. Otolaryngologists are often the first place KFS patients are referred, as they manage patients with commonly associated issues related to KFS including hearing loss, dysphagia, and craniofacial issues. Further, KFS frequently presents with ear, nose, and throat (ENT)-related symptoms such as hearing loss, dysphagia, cleft palate, jaw disorders, ear malformation, and airway obstruction. Together these symptoms make an education and knowledge of KFS highly relevant in the field of otolaryngology. This paper will examine cases and outcomes of KFS seen in the last 10 years to increase the knowledge of KFS specifically in the field of otolaryngology. As far as the authors are aware, this is the most in-depth analysis of KFS in the field of otolaryngology.

## Review

Methods

This is a systematic review of case reports related to KFS published in the last 10 years in the PubMed database. The initial search retrieved case reports with the keyword “Klippel-Feil Syndrome". Subsequent filtering was carried out using the keywords “hearing loss”, “dysphagia”, “retrognathia”, “micrognathia”, “cleft lip”, “cleft palate”, “thyroid”, “ear”, “mallampati”, and “cormack-lehane”. The inclusion criteria for this systematic review were each case that included the otolaryngological symptoms of hearing loss, dysphagia, jaw malformations, cleft lip or palate, thyroid malformations, or reported a Mallampati or Cormack-Lehane score. Case reports excluded were those that did not present with these specific otolaryngological symptoms or were not human subjects.

**Figure 1:**
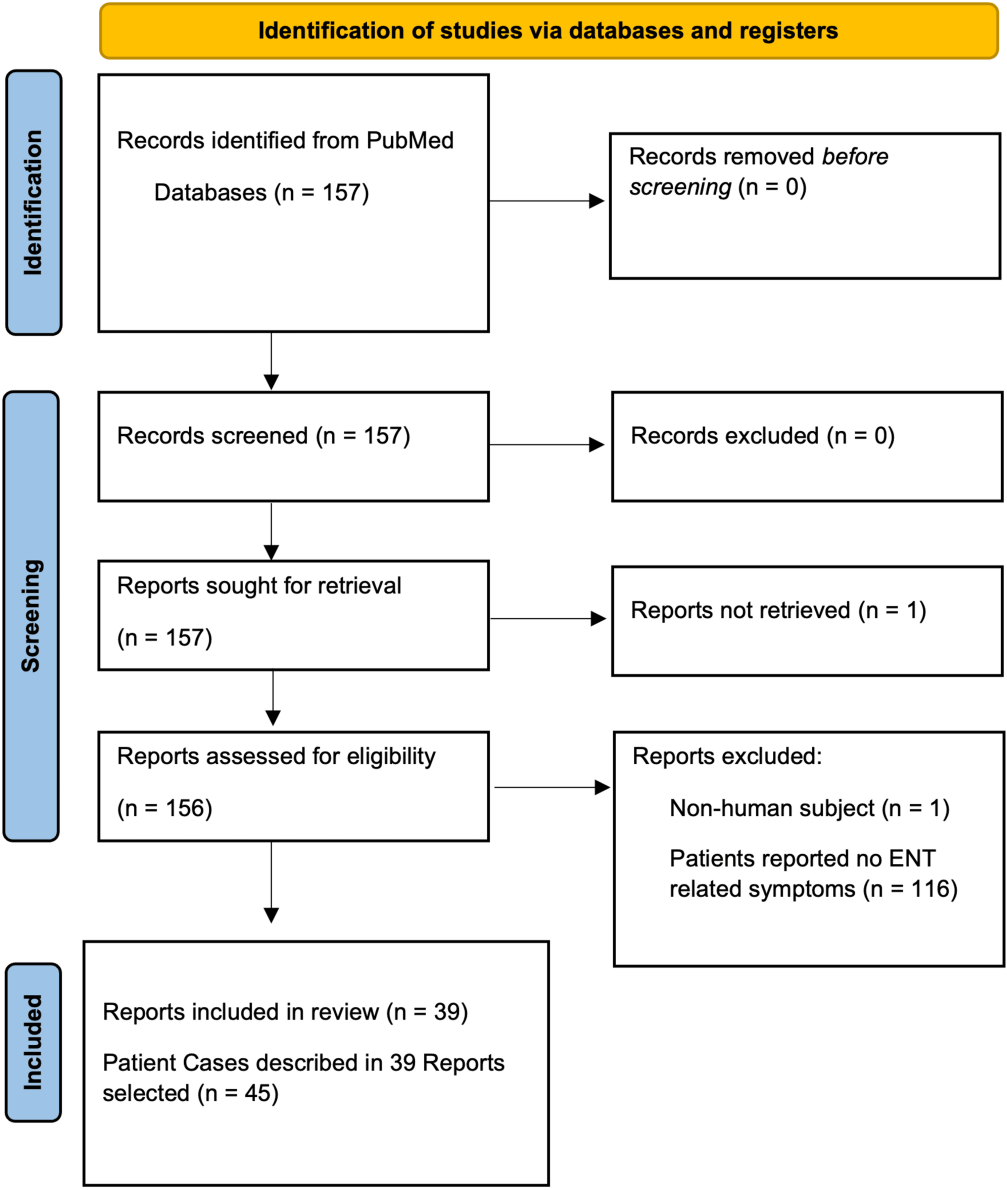
Preferred Reporting Items for Systematic Reviews and Meta-Analyses (PRISMA) Diagram

Each of these cases was carefully analyzed and compared using the Samartzis classification scale. This process can be seen in the Preferred Reporting Items for Systematic Reviews and Meta-Analyses (PRISMA) diagram found in Figure 1.

Each of these cases was then carefully analyzed independently by two reviewers. Four reviewers then collaborated to group cases using the Samartzis classification scale along with the Mallampati and Cormack-Lehane scales. Cases were then grouped based on the accompanying symptoms, and the types of imaging that were obtained to help physicians understand KFS. Patients were compared based on their Samartzis classification, Mallampati grade, and Cormack-Lehane score.

The Samartzis classification was developed in 2006 and groups KFS patients based on the type of spinal cord fusion seen [9]. Patients who have only a single fusion in the cervical region are classified as type 1, patients with multiple non-contiguous fusions are type 2, and multiple fused segments in the cervical region are classified as type 3 [9]. Patients with type 1 fusions tend to have symptoms that are more axial whereas types 2 and 3 tend to have symptoms of radiculopathy and myelopathy [9].

Once patients were grouped based on Samartzis classification, the specific otolaryngological symptoms of hearing loss, jaw disorders, cleft palate, thyroid malformation, dysphagia, and ear malformations were carefully analyzed. Along with each of these symptoms, articles were screened to identify other affected body systems based on the International Classification of Diseases, Tenth Revision (ICD-10) codes to identify other patient care needs that may require evaluation, or refer to other specialists for more comprehensive care [10]. The ICD-10 codes were used to categorize the symptoms that accompanied different fusions.

The Mallampati score system and Cormack-Lehane grading systems were also analyzed and compared in order to make conclusions based on airway management in KFS patients. The Mallampati grading system looks at the visibility of structures in the oropharynx, and the Cormack-Lehane grading system looks at the visibility of the glottis and other airway structures [11,12]. Both of these are graded on a scale from 1 to 4, with an increased number being associated with a more closed airway, and likely a more challenging intubation [11,12]. This is important for predicting intubation success and airway management for often-needed surgeries in KFS patients.

The purpose of this study is to identify correlations between different classes of spinal cord fusions and specific symptoms, as well as to identify the best practices for the management of patients with KFS. Data acquired was analyzed using descriptive statistics and then presented in tables and pie charts.

Quality Assessment and Risk of Bias

The Joanna Briggs Institute Criteria for Case Reports (JBI) was applied to all case reports utilized in this systematic review [13]. JBI analyzes the quality of the case reports by looking at the following criteria: clear description of patient demographics, patient history, clinical condition, diagnostic tests, intervention, post-intervention follow-up, patient safety, and key takeaway messages [13]. To ensure the accuracy of the data, the JBI test was utilized. All case reports in this study adhered to the JBI criteria and none were removed from the study. While the risk of bias in this study is considered to be low, reporting bias is a definite possibility. KFS case reports were written by various specialists who all have a different focus and training. Each case had a different focus based on the specialty of the writer that impacted the report. In addition, none of the articles utilized declared any conflict of interest or had any financial disclosures.

Results

A total of 44 cases with reported ENT abnormalities were analyzed. (There was one patient that reported a Mallampati score but had no abnormalities.) Table 1 contains the data collected from patients who presented with hearing loss.

**Table 1:** KFS Patients With Hearing Symptoms The classic triad includes a low posterior hairline, short neck, and limited cervical range of motion [2]. KFS: Klippel-Feil syndrome, ROM: range of motion, CT: computed tomography, US: ultrasound, MRI: magnetic resonance imaging, BLOOD: Diseases of the blood and blood-forming organs and certain disorders involving the immune mechanism, ENDO: Endocrine, nutritional and metabolic diseases, CNS: Mental, Behavioral and Neurodevelopmental disorders, NEURO: Diseases of the Nervous System, EYE: Diseases of the eye and adnexa, EAR: Diseases of the ear and mastoid process, CIR: Diseases of the Circulatory System, RESP: Diseases of the respiratory system, GI: Diseases of the digestive system, INT: Diseases of the skin and subcutaneous tissue, MSK: Diseases of the musculoskeletal system and connective tissue, GU: Diseases of the genitourinary system, PREG: Pregnancy, childbirth, and the puerperium, GEN: Congenital malformations, deformations, and chromosomal abnormalities, OTHER: Symptoms, signs, and abnormal clinical and laboratory findings, not elsewhere classified

Author	Age (Years Old) and Gender M (Male) or F (Female)	Hearing-Related Symptoms	Comorbidities and Characteristics	Cervical Fusion	Samartzis Classification	Classic Triad: Y(Yes) or N (No)	Imaging
Carruyo et al., 2016 [14]	11 y/o M	Right-sided hearing loss.	Limited cervical ROM (MSK), low posterior hairline (INT).	Partial C4-C5, Complete C5-C6	Type 3	N	Not listed
Singh et al., 2019 [15]	2 day old M	Bilateral sensorineural hearing loss confirmed by audiometry.	Constipation (GI), low posterior hairline (INT), anorectal malformation (GEN), solitary kidney (GEN), Sprengel deformity (GEN), limited cervical ROM (MSK), short neck (GEN).	Cervical Fusion present.	Not listed	Y	X-ray, US, micturating cystogram
Alaqeel, 2014 [16]	5 day old F	Bilateral sensorineural hearing loss. Patient born deaf. Cochlear dysplasia found upon imaging.	High scapula (GEN), short neck (GEN), low posterior hairline (INT). Mondini malformation (GEN), ASD (CIR).	C5-C7 Fusion	Type 3	N	CT, MRI, US (kidney)
Ishida et al., 2017 [17]	75 y/o F	Hearing loss since childhood.	Intraventricular hemorrhage (NEURO), gait (NEURO), hydrocephalus (enlarged lateral, 3rd, and 4th ventricles) (NEURO), short neck (GEN), low posterior hairline (INT)	C2-C4 and C5-C7	Type 3	N	MRI
Chima Galan et al., 2022 [18]	14 y/o F	Left-sided deafness. Fusion of the bony labyrinth with vestigial remnants of the semicircular canals and cochlea.	Left hemifacial microsomia (GEN), wide nasal bridge (MSK), limited bilateral eye ROM (NEURO), short neck (GEN), limited cervical ROM (MSK), short thorax (GEN), scoliosis (GEN), camptodactyly (GEN), Duane syndrome (GEN), strabismus (GEN), mitral valve prolapse (CIR), Wildervanck syndrome (GEN).	C4-C5	Type 1	N	CT, ECG, US (kidney)
Roberti et al., 2018 [19]	22 y/o F	Bilateral mixed hearing loss.	Micrognathia (GEN), glossoptosis (GEN), cleft palate (GEN), triphalangeal thumbs (GEN), hyporegenerative anemia (BLOOD), prominent/abnormal facial structures (GEN), ocular asymmetry with bupthalmos (EYE), exotropia (GEN), external acoustic meatus malformity (GEN), tooth decay and cavities (GI), scoliosis (GEN), clinodactyly of the fingers (GEN), hip dysmetria (OTHER), Pierre Robin sequence (GEN), neurodevelopmental delay (CNS), neck pain (MSK), perimembraneous ventricular defect (GEN), trigeminal nerve palsy (NEURO), rhinolalia (GEN), dysarthria (OTHER), dysphagia (OTHER), short neck (GEN).	Atlanto-occipital joint fusion, C2-C3, C4-C5, L4-L5	Type 3	N	CT, MRI
Jamrozik et al., 2015 [20]	68 y/o M	Hearing loss	Dysarthria (OTHER), low cognitive function (CNS), jaw jerk (NEURO), increased gag reflex (NEURO), palmomental reflex (NEURO), pathological crying (CNS), tongue atrophy and fasciculations (NEURO), short neck (GEN), low posterior hairline (INT), muscle fasciculations (NEURO), widened central spinal canal in cervical and thoracic vertebrae (GEN), ALS (NEURO), Chiari type 1 malformation (GEN), syringomyelia (NEURO).	C2-C3	Type 1	N	CT, MRI
Aravindan et al., 2021 [21]	38 y/o F	Ear discharge and sensorineural hearing loss in the left ear.	Short neck (GEN), webbed neck (GEN), limited cervical ROM (MSK), low posterior hairline (INT), cleft lip (GEN).	C1-C3	Type 3	Y	Not Listed
Aravindan et al., 2021 [21]	18 y/o F	Sensorineural unilateral hearing loss.	Short neck (GEN), webbed neck (GEN), low posterior hairline (INT), limited cervical ROM (MSK), bifid uvula (GEN), prognathism (GEN), pectus carinatum (GEN), thumb hypoplasia (GEN), absent metacarpal (GEN), intellectual disability (CNS), delayed sexual characteristics (ENDO).	C2-C7	Type 3	Y	Not LIsted
Abukabbos et al., 2015 [22]	13 y/o F	Hearing loss and chronic otitis media.	Restricted mouth opening (MSK), VACTERL (GEN), right kidney agenesis (GEN), vesicoureteral reflux (GU), Sprengel deformity (GEN), Syringomyelia (NEURO), Chiari Malformation (GEN), Restrictive lung disease (RESP), T&A hypertrophy (RESP), asthma (RESP), obstructive sleep apnea (RESP), esophageal atrasia (GEN), gastroesophageal reflux disease (GI), upper limb congenital malformation (GEN), heart defect (GEN), short neck (GEN), torticollis (MSK), webbed neck (GEN).	Not listed.	Not listed	N	Not Listed
Jasper et al., 2014 [23]	11 y/o F	Hearing loss in left ear. Features of type I incomplete partition were seen in the left inner ear with a cystic-appearing cochlea lacking the entire modiolus and cribriform area and a large cystic vestibule. Lateral semicircular canal angle with absent central bone island was also seen.	Aberrant right subclavian artery (CIR), cervicomedullary neuroschisis (GEN), wide foramen magnum (GEN).	Multiple cervical and posterior arch fusions.	Not listed	N	MRI, CT
Sabol et al., 2014 [24]	51 y/o F	Hearing loss.	Shortness of breath (RESP), asymmetry in brachial pulse, scoliosis (GEN), renal insufficiency (GU), severe heart defects (CIR).	Not listed	Not listed	N	CT
Giampietro et al., 2014 [25]	No age listed, M	Bilateral conductive hearing loss.	Limited cervical and shoulder ROM (MSK), short neck (GEN).	C2-C4	Type 3	N	X-ray
Giampietro et al., 2014 [25]	8 y/o F	Unilateral sensorineural hearing loss.	Limited cervical ROM (MSK), low posterior hairline (INT), short neck (GEN).	C2-C4, C5-C6, C7-T1	Type 3	Y	X-ray
Giampietro et al., 2014 [25]	0 y/o F	Bilateral conductive deafness at birth.	None listed.	C2-C5, T3-T4	Type 3	N	Not listed
Sekeroglu et al., 2014 [26]	13 y/o F	Conductive hearing loss confirmed by audiometry.	Inward deviation of the left eye (EYE), short neck (GEN), webbed neck (GEN), limited cervical ROM (MSK), facial asymmetry (GEN), intellectual disability (CNS), Wildervanck Syndrome (GEN).	C1-C7,	Type 3	N	MRI
Hernando et al., 2014 [27]	1 y/o M	Right ear profound sensorineural hearing loss, confirmed with BERA (Brainstem Evoked Response Audiometry) and behavioral observation audiology, and normal hearing in left ear. A complex right inner ear malformation was shown. MRI imaging showed a narrowing of the right internal auditory canal, with small caliber of cisternal routes of VII and VIII cranial nerves, and no evidence of the right cochlea. Only rudiments of superior semicircular canal and vestibule could be identified.	Dysmorphic facial features (GEN), Duane syndrome (GEN), heart malformation (CIR), arterial malformations (CIR).	Occipitocervical junction	Type 1	N	MRI, US (heart), CT
Duggal et al., 2022 [28]	16 y/o M	Hearing loss and pain in the right ear. Discharge from the ear.	Cleft lip and palate (GEN), neck pain (MSK), misaligned jaw (GEN), short neck (GEN), low posterior hairline (INT), limited cervical ROM (MSK), torticollis (MSK), sprengel deformity (GEN), maxillary hypoplasia (GEN), TMJ (MSK), deviated septum (GEN), irregular teeth formation (GI), missing teeth (GI), restricted mouth opening (MSK), TMJ (MSK).	C3-C7	Type 3	Y	CT, X-Ray, PA Cephalograms
Sirico et al., 2023 [29]	34 y/o F	Bilateral deafness managed with endoauricular hearing aids.	Short neck (GEN), low posterior hairline (INT), limited cervical ROM (MSK), scoliosis (GEN), Sprengel deformity (GEN).	Atlanto-occipital joint, C2-C3	Type 1	Y	MRI
Minatogawa et al., 2021 [30]	59 y/o M	Bilateral high-frequency sensorineural hearing impairment and left moderate conductive hearing impairment with thresholds of 53.8 dB and 25 dB in the left and right ears, respectively. Right conductive hearing impairment was caused by the decreased mobility of the tympanic membrane associated with cleft palate.	Paralysis, Cleft palate (GEN), syndactyly of the toes (GEN), dysarthria (OTHER), mastication difficulty (MSK), prognathism (GEN), hypoplastic maxillary bone (GEN), Elsahy–Waters syndrome (GEN).	C2-C3	Type 1	N	X-Ray
Carruyo et al., 2016 [14]	16 y/o M	Unilateral hearing loss. Auricle and external auditory canal agenesis.	Tracheoesophageal fistula (GEN), cervical deformity (GEN), limited cervical ROM (MSK), occipital hair loss (INT), Sprengel’s deformity (GEN), right bifid thumb due to accessory distal phalanx (GEN), unilateral renal agenesis (GEN).	C4-C5, C5-C6	Type 3	N	X-ray

Table 2 contains the patients who presented with jaw symptoms.

**Table 2:** KFS Patients With Jaw Symptoms The classic triad includes a low posterior hairline, short neck, and limited cervical range of motion [2]. KFS: Klippel-Feil syndrome, ROM: range of motion, CT: computed tomography, US: ultrasound, MRI: magnetic resonance imaging, BLOOD: Diseases of the blood and blood-forming organs and certain disorders involving the immune mechanism, ENDO: Endocrine, nutritional and metabolic diseases, CNS: Mental, Behavioral and Neurodevelopmental disorders, NEURO: Diseases of the Nervous System, EYE: Diseases of the eye and adnexa, EAR: Diseases of the ear and mastoid process, CIR: Diseases of the Circulatory System, RESP: Diseases of the respiratory system, GI: Diseases of the digestive system, INT: Diseases of the skin and subcutaneous tissue, MSK: Diseases of the musculoskeletal system and connective tissue, GU: Diseases of the genitourinary system, PREG: Pregnancy, childbirth, and the puerperium, GEN: Congenital malformations, deformations, and chromosomal abnormalities, OTHER: Symptoms, signs, and abnormal clinical and laboratory findings, not elsewhere classified

Author	Age (Years Old) and Gender M (Male) or F (Female)	Jaw-Related Symptoms	Comorbidities and Characteristics	Cervical Fusion	Samartzis Classification	Classic Triad: Y(Yes) or N (No)	Imaging
Nuanay et al., 2019 [31]	5 y/o F	Retrognathia	Scoliosis (GEN), short neck (GEN), low posterior hairline (INT), limited cervical ROM (MSK), hemivertebrae (GEN), limited range of motion of extremities (MSK), fusion of carpal bones (GEN), missing rib 12 (GEN).	C1-C3, C4-C5, C6-C7	Type 3	Y	CT
Roberti et al., 2018 [19]	22 y/o F	Micrognathia	Bilateral mixed hearing loss (NEURO), glossoptosis (GEN), cleft palate (GEN), triphalangeal thumbs (GEN), hyporegenerative anemia (BLOOD), prominent/abnormal facial structures (GEN), ocular asymmetry with buphthalmos (GEN), exotropia (EYE), external acoustic meatus malformity (EAR), tooth decay and cavities (GI), scoliosis (GEN), clinodactyly of the fingers (GEN), hip dysmetria (OTHER), Pierre Robin sequence (GEN), neurodevelopmental delay (CNS), neck pain (MSK), perimembraneous ventricular defect (CIR), trigeminal nerve palsy (NEURO), rhinolalia (GEN), dysarthria (OTHER), dysphagia (OTHER), short neck (GEN).	Atlanto-occipital joint fusion, C2-C3, C4-C5, L4-L5	Type 3	N	CT, MRI
Schieffer et al., 2019 [32]	4 y/o M	Micrognathia	Dysphagia (OTHER), developmental delays (CNS), blue sclera (EYE), high arched palate (GEN), scoliosis (GEN), hip dysplasia (GEN), obstructive hydrocephalus due to mass in 4th ventricle (NEURO), group 3ᵧ medulloblastoma (NEURO).	C2-C3	Type 1	N	MRI
Alazami et al., 2014 [33]	12 y/o F	Micrognathia	Facial deformity evident at birth (GEN), short neck (GEN), hirsutism (ENDO), low posterior hairline (INT), low set ears (GEN), bilateral ptosis (EYE), high arched palate (GEN), webbed neck (GEN), myopathy (MSK).	Cervical spinal fusion	Not listed	N	X-Ray
Aravindan et al., 2021 [21]	18 y/o F	Prognathism	Unilateral hearing loss (NEURO), snoring (RESP), short neck (GEN), webbed neck (GEN), limited cervical ROM (MSK), low posterior hairline (INT), large tongue (GEN), bifid uvula (GEN), pectus carinatum (GEN), left thumb hypoplasia (GEN), absent metacarpal (GEN), intellectual disability (CNS), delayed sexual characteristics (ENDO), chronic otitis media (EAR), Mayer Rokitansky Kuster Hauser Syndrome (GEN).	C2-C7	Type 3	Y	Not Listed
Hase et al., 2014 [34]	27 y/o F	Skeletal maxillary protrusion with mandibular retrognathia	Limited cervical ROM (MSK), asymmetry of clavicle and scapula (GEN), limited arm ROM (MSK), deviated nasal septum (GEN).	C3-C5	Type 3	N	X-ray
Paramaswamy, 2014 [35]	16 y/o M	Prognathism	Limited cervical ROM (MSK), low posterior hairline (INT), misaligned teeth (GI), overbite (GI), psychological disturbances (NEURO), kyphoscoliosis (GEN).	C1-C6	Type 3	Y	X-ray, CT
Pagano et al., 2019 [36]	70 y/o M	Prognathism	Scoliosis (GEN), airway management difficulties (GEN).	Not listed	Not listed	N	CT

Table 3 contains all patients who presented with cleft lip or cleft palate.

**Table 3:** KFS Patients With Cleft Lip or Palate The classic triad includes a low posterior hairline, short neck, and limited cervical range of motion [2]. KFS: Klippel-Feil syndrome, ROM: range of motion, CT: computed tomography, US: ultrasound, MRI: magnetic resonance imaging, BLOOD: Diseases of the blood and blood-forming organs and certain disorders involving the immune mechanism, ENDO: Endocrine, nutritional and metabolic diseases, CNS: Mental, Behavioral and Neurodevelopmental disorders, NEURO: Diseases of the Nervous System, EYE: Diseases of the eye and adnexa, EAR: Diseases of the ear and mastoid process, CIR: Diseases of the Circulatory System, RESP: Diseases of the respiratory system, GI: Diseases of the digestive system, INT: Diseases of the skin and subcutaneous tissue, MSK: Diseases of the musculoskeletal system and connective tissue, GU: Diseases of the genitourinary system, PREG: Pregnancy, childbirth, and the puerperium, GEN: Congenital malformations, deformations, and chromosomal abnormalities, OTHER: Symptoms, signs, and abnormal clinical and laboratory findings, not elsewhere classified

Author	Age (Years Old) and Gender M (Male) or F (Female)	Cleft Palate	Comorbidities and Characteristics	Cervical Fusion	Samartzis Classification	Classic Triad: Y(Yes) or N (No)	Imaging
Bhat et al., 2014 [37]	1 y/o F	Cleft Palate	Thoracic kyphosis (MSK), hypoplastic C3 and C6 vertebrae (GEN), short neck (GEN), limited cervical ROM (MSK), occipital meningocele (NEURO).	Hypoplastic C3 and C6	Not listed	Y	X-Ray, CT
Roberti et al., 2018 [19]	22 y/o F	Cleft Palate	Bilateral mixed hearing loss (NEURO), glossoptosis (GEN), cleft palate (GEN), triphalangeal thumbs (GEN), hyporegenerative anemia (BLOOD), prominent/abnormal facial structures (GEN), ocular asymmetry with buphthalmos, exotropia (GEN), external acoustic meatus malformity (EAR), tooth decay and cavities (GI), scoliosis (GEN), clinodactyly of the fingers (GEN), hip dysmetria (OTHER), Pierre Robin sequence (GEN), neurodevelopmental delay (CNS), neck pain (MSK), perimembraneous ventricular defect (CIR), trigeminal nerve palsy (NEURO), rhinolalia (GEN), dysarthria (OTHER), dysphagia (OTHER), short neck (GEN).	Atlanto-occipital joint fusion, C2-C3, C4-C5, L4-L5	Type 3	N	CT, MRI
Edward et al., 2017 [38]	38 y/o F	Cleft lip and palate	Nasal cavity mass (INT), headaches (NEURO), nausea (OTHER), vomiting (OTHER), nasal obstruction (OTHER), webbing of the neck (GEN).	C2-C3 fusion	Type 1	N	CT, MRI, endoscopy
Kavanaugh et al., 2013 [39]	38 y/o F	Cleft palate	Chronic neck and back pain (MSK), cervical spina bifida cystica myelomeningocele (NEURO), multiple cleft vertebrae (GEN), kyphoscoliosis (GEN), limited mouth opening (MSK), short neck (GEN), limited cervical ROM (MSK), thoracic and lumbar scoliosis (GEN), Sprengel deformity (GEN), webbed neck (GEN), large foramen magnum (GEN).	C2-C4, C5-C7, T3-T5	Type 3	N	MRI
Aravindan et al., 2021 [21]	38 y/o F	Cleft lip	Short neck (GEN), webbed neck (GEN), limited cervical ROM (MSK), low posterior hairline (INT), ear discharge (EAR), sensorineural hearing loss (NEURO).	C1-C3	Type 3	Y	Not listed
Duggal et al., 2022 [28]	16 y/o M	Complete unilateral cleft lip and palate	Hearing loss (NEURO), unilateral ear pain (EAR), discharge from ear (EAR), neck pain (MSK), misaligned jaw (GEN), short neck (GEN), low posterior hairline (INT), limited cervical ROM (MSK), torticollis (MSK), sprengel deformity (GEN), maxillary hypoplasia (GEN), TMJ (MSK), deviated septum (GEN), irregular teeth formation (GI), missing teeth (GI), restricted mouth opening (MSK), TMJ (MSK).	C3-C7	Type 3	Y	CT, X-ray, cephalogram
Xu et al., 2021 [40]	41 y/o F	Cleft lip	Intellectual disability (CNS), substance abuse (CNS), anxiety (CNS), scoliosis (GEN), C2-C4 vertebral canal stenosis and retrolisthesis (GEN), urinary retention (GU).	C4-C5	Type 1	N	MRI, X-ray, CT
Munter et al., 2014 [41]	28 y/o F	Cleft Palate	abnormal face (GEN), sprengel deformity (GEN), scoliosis (GEN), C3-C4 stenosis (GEN), two instances of sudden loss of all strength in all 4 extremities (NEURO).	C2-C3	Type 1	N	ECG

Table 4 contains all patients who presented with thyroid symptoms.

**Table 4:** KFS Patients With Thyroid Symptoms The classic triad includes a low posterior hairline, short neck, and limited cervical range of motion [2]. KFS: Klippel-Feil syndrome, ROM: range of motion, CT: computed tomography, US: ultrasound, MRI: magnetic resonance imaging, BLOOD: Diseases of the blood and blood-forming organs and certain disorders involving the immune mechanism, ENDO: Endocrine, nutritional and metabolic diseases, CNS: Mental, Behavioral and Neurodevelopmental disorders, NEURO: Diseases of the Nervous System, EYE: Diseases of the eye and adnexa, EAR: Diseases of the ear and mastoid process, CIR: Diseases of the Circulatory System, RESP: Diseases of the respiratory system, GI: Diseases of the digestive system, INT: Diseases of the skin and subcutaneous tissue, MSK: Diseases of the musculoskeletal system and connective tissue, GU: Diseases of the genitourinary system, PREG: Pregnancy, childbirth, and the puerperium, GEN: Congenital malformations, deformations, and chromosomal abnormalities, OTHER: Symptoms, signs, and abnormal clinical and laboratory findings, not elsewhere classified

Author	Age (Years Old) and Gender M (Male) or F (Female)	Thyroid-Related Symptoms	Comorbidities and Characteristics	Cervical Fusion	Samartzis Classification	Classic Triad: Y(Yes) or N (No)	Imaging
Santonastaso et al., 2019 [42]	26 y/o F	Hypothyroidism	Dystocia (PREG), solitary kidney (GEN), chronic neck pain (MSK), pineal cyst (NEURO), Rathke’s cleft cyst (ENDO), hypertension, scoliosis (GEN), short neck (GEN), limited cervical ROM (MSK).	Not Listed	Not listed	N	X-ray
Strand et al., 2016 [43]	34 y/o F	Hashimoto encephalopathy, severe hypothyroidism	Dystonia of the extremities (MSK), severe jaw dystonia (MSK), neuropathic pain (NEURO).	C5-C6	Type 1	N	Not listed

Table 5 contains all patients who presented with dysphagia.

**Table 5:** KFS Patients With Dysphagia The classic triad includes a low posterior hairline, short neck, and limited cervical range of motion [2]. KFS: Klippel-Feil syndrome, ROM: range of motion, CT: computed tomography, US: ultrasound, MRI: magnetic resonance imaging, BLOOD: Diseases of the blood and blood-forming organs and certain disorders involving the immune mechanism, ENDO: Endocrine, nutritional and metabolic diseases, CNS: Mental, Behavioral and Neurodevelopmental disorders, NEURO: Diseases of the Nervous System, EYE: Diseases of the eye and adnexa, EAR: Diseases of the ear and mastoid process, CIR: Diseases of the Circulatory System, RESP: Diseases of the respiratory system, GI: Diseases of the digestive system, INT: Diseases of the skin and subcutaneous tissue, MSK: Diseases of the musculoskeletal system and connective tissue, GU: Diseases of the genitourinary system, PREG: Pregnancy, childbirth, and the puerperium, GEN: Congenital malformations, deformations, and chromosomal abnormalities, OTHER: Symptoms, signs, and abnormal clinical and laboratory findings, not elsewhere classified

Author	Age (Years Old) and Gender M (Male) or F (Female)	Dysphagia-Related Symptoms	Comorbidities and Characteristics	Cervical Fusion	Samartzis Classification	Classic Triad: Y(Yes) or N (No)	Imaging
Roberti et al., 2018 [19]	22 y/o F	Dysphagia	Bilateral mixed hearing loss (NEURO), glossoptosis (GEN), cleft palate (GEN), triphalangeal thumbs (GEN), hyporegenerative anemia (BLOOD), prominent/abnormal facial structures (GEN), ocular asymmetry with buphthalmos (GEN), exotropia (GEN), external acoustic meatus malformity (GI), tooth decay and cavities (GI), scoliosis (GEN), clinodactyly of the fingers (GEN), hip dysmetria (OTHER), Pierre Robin sequence (GEN), neurodevelopmental delay (CNS), neck pain (MSK), perimembraneous ventricular defect (CIR), trigeminal nerve palsy (NEURO), rhinolalia (GEN), dysarthria (OTHER), short neck (GEN), cleft palate (GEN)	Atlanto-occipital joint fusion, C2-C3, C4-C5, L4-L5	Type 3	N	CT, MRI
Clarke et al., 2021 [44]	19 y/o M	Severe dysphagia, microstomia making it hard to swallow, overcrowded teeth	Severe speech impairment (CNS), GDF6 gain of function mutation (GEN), multiple stenosis syndrome (GEN), thinning of space between C6-C7 (GEN)	C2-C3, C4-C5	Type 3	N	X-ray
Tian et al., 2018 [45]	12 y/o F	Dysphagia, choking	LE weakness (MSK), hoarseness (OTHER), hyperreflexia in LE (NEURO), positive Hoffman’s sign b/l (NEURO), unsteady gait (NEURO), limited cervical ROM (MSK), stenosis (NEURO), upward migration of odontoid process (GEN),	C1 occipitalization,. C2-C3, C6-C7,	Type 3	N	MRI, CT, X-ray
Pacca et al., 2019 [46]	69 y/o M	Dysphagia	Dysphonia (OTHER), balance issues (OTHER), vomiting, hyperreflexia in LE (NEURO), tetraparesis (NEURO), positive cutaneous plantar reflex b/l (NEURO), cystic lesion in brain stem (NEURO), CVJ stenosis (GEN), syringobulbia (NEURO), cervical instability (MSK)		Not Listed	N	CT, MRI
Pacreu et al., 2018 [47]	43 y/o M	Paresthesia of tongue and palate, difficulty opening mouth, limited mandible subluxation	Nystagmus (EYE), facial hemianesthesia (GEN), dysphonia (OTHER), syringomyelia (NEURO), limited cervical ROM (MSK), low posterior hairline (INT), short neck (GEN), facial dysmorphism (GEN), Chiari disease (NEURO),	Fusion of cervical vertebrae	Not Listed	Y	MRI

Table 6 contains all patients who presented with ear malformations.

**Table 6:** Hearing Malformations in KFS Patients The classic triad includes a low posterior hairline, short neck, and limited cervical range of motion [2]. KFS: Klippel-Feil syndrome, ROM: range of motion, CT: computed tomography, US: ultrasound, MRI: magnetic resonance imaging, BLOOD: Diseases of the blood and blood-forming organs and certain disorders involving the immune mechanism, ENDO: Endocrine, nutritional and metabolic diseases, CNS: Mental, Behavioral and Neurodevelopmental disorders, NEURO: Diseases of the Nervous System, EYE: Diseases of the eye and adnexa, EAR: Diseases of the ear and mastoid process, CIR: Diseases of the Circulatory System, RESP: Diseases of the respiratory system, GI: Diseases of the digestive system, INT: Diseases of the skin and subcutaneous tissue, MSK: Diseases of the musculoskeletal system and connective tissue, GU: Diseases of the genitourinary system, PREG: Pregnancy, childbirth, and the puerperium, GEN: Congenital malformations, deformations, and chromosomal abnormalities, OTHER: Symptoms, signs, and abnormal clinical and laboratory findings, not elsewhere classified

Author	Age (Years Old) and Gender M (Male) or F (Female)	Ear Malformation	Comorbidities and Characteristics	Cervical Fusion	Samartzis Classification	Classic Triad: Y(Yes) or N (No)	Imaging
Chima Galan et al., 2022 [18]	14 y/o F	Left sided deafness. Fusion of the bony labyrinth with vestigial remnants of the semicircular canals and cochlea.	Left hemifacial microsomia (GEN), wide nasal bridge (MSK), limited bilateral eye ROM (NEURO), short neck (GEN), limited cervical ROM (MSK), short thorax (GEN), scoliosis (GEN), camptodactyly (GEN), Duane syndrome (GEN), strabismus (GEN), mitral valve prolapse (CIR), Wildervanck syndrome (GEN),	C4-C5	Type 1	N	CT, ECG, US (kidney)
Hernando et al., 2014 [27]	1 y/o M	Right ear profound sensorineural hearing loss, confirmed with BERA (Brainstem Evoked Response Audiometry) and behavioral observation audiology, and normal hearing in left ear. A complex right inner ear malformation was shown. MRI imaging showed a narrowing of the right IAC, with small caliber of cisternal routes of VII and VIII cranial nerves, and no evidence of the right cochlea. Only rudiments of superior semicircular canal and vestibule could be identified.	Dysmorphic facial features (GEN), Duane syndrome (GEN), heart malformation (GEN), arterial malformations (CIR)	Occipitocervical junction	Type 1	N	MRI, US (heart), CT
Alaqeel et al., 2014 [16]	5 day old F	Bilateral sensorineural hearing loss. Patient born deaf. Cochlear dysplasia found upon imaging.	High scapula (GEN), short neck (GEN), low posterior hairline (INT). Mondini malformation (GEN), ASD (CIR)	C5-C7 Fusion	Type 3	N	CT, MRI, US (kidney)
Jasper et al., 2014 [23]	11 y/o F	Hearing loss in left ear. Features of type I incomplete partition were seen in the left inner ear with a cystic-appearing cochlea lacking entire modiolus and cribriform area and a large cystic vestibule. Lateral semicircular canal anlage with absent central bone island was also seen.	Aberrant right subclavian artery (CIR). Cervicomedullary neuroschisis (GEN), wide foramen magnum (GEN)	Multiple cervical and posterior arch fusions.	Not Listed	N	MRI, CT
Minatogawa et al., 2021 [30]	59 y/o M	bilateral high-frequency sensorineural hearing impairment and left moderate conductive hearing impairment with thresholds of 53.8 dB and 25 dB in the left and right ears, respectively. Right conductive hearing impairment was caused by the decreased mobility of the tympanic membrane associated with cleft palate.	Paralysis, Cleft palate (GEN), syndactyly of the toes (GEN), dysarthria (OTHER), mastication difficulty (MSK), prognathism (GEN), hypoplastic maxillary bone (GEN), Elsahy–Waters syndrome (GEN)	C2-C3	Type 1	N	X-Ray
Carruyo et al., 2016 [14]	16 y/o M	Unilateral hearing loss. Auricle and external auditory canal agenesis,	Tracheoesophageal fistula (GEN), cervical deformity (GEN), limited cervical ROM (MSK), occipital hair loss (INT), Sprengel’s deformity (GEN), right bifid thumb due to accessory distal phalanx (GEN), unilateral renal agenesis (GEN),	C4-C5, C5-C6	Type 3	N	X-ray

Demographics of KFS patients and stratification by Samartzis class are found in Table 7. Overall statistics are provided, as well as a breakdown of each ENT-related symptom. The number of patients that presented with the classic triad (low posterior hairline, short neck, and limited cervical range of motion) is also reported.

**Table 7:** Demographics and Samartzis Classification of Klippel-Feil Syndrome Patients N: number of cases; SD: standard deviation. * Classic triad represents a short neck, low posterior hairline, and limited cervical range of motion [2]. ** includes Mallampati and Cormack-Lehane patients in Table 4.

Symptom	Sample Size	Gender N (%)		Age (Years) Mean (SD)	Samartzis Classification: N (%)				Presenting With Classic Triad*: N (%)
		Male	Female		Type 1	Type 2	Type 3	Not Listed	
Hearing loss: [14-30]	21 cases	8 (38%)	13 (62%)	23.35 (23.15)	5 (24%)	0 (0%)	12 (57%)	4 (19%)	6 (29%)
Jaw Disorders: [19,21,31-36]	8 cases	3 (37%)	5 (63%)	21.75 (21.02)	1 (12%)	0 (0%)	5 (63%)	2 (25%)	3 (38%)
Cleft Lip/Palate: [19,21,28,37-41]	8 cases	1 (12%)	7 (88%)	27.75 (14.03)	3 (37.5%)	0 (0%)	4 (50%)	1 (12.5%)	3 (38%)
Thyroid: [42,43]	2 cases	0 (0%)	2 (100%)	30 (5.66)	1 (50%)	0 (0%)	0 (0%)	1 (50%)	0 (0%)
Dysphagia: [19,44-47]	5 cases	3 (60%)	2 (40%)	33 (23.21)	0 (0%)	0 (0%)	3 (60%)	2 (40%)	1 (20%)
Ear Deformity: [14,16,18,23,27,30]	6 cases	3 (50%)	3 (50%)	16.67 (21.85)	3 (50%)	0 (0%)	2 (33%)	1 (17%)	0 (0%)
Overall Statistics**	44 cases	16 (36%)	28 (64%)	24.3 (20.75)	11 (25%)	0 (0%)	21 (48%)	12 (27%)	12 (28%)

Next, the associated symptoms were analyzed for each symptom (Table 8). Associated symptoms were reported by individual symptoms and overall statistics. It is important to note that many patients received more than one symptom. 

**Table 8:** Klippel-Feil Syndrome Symptoms Stratified by International Classification of Diseases, Tenth Revision (ICD-10) Codes N: number of cases

Associated Symptoms or Comorbidities: N (%)	Congenital Malformations, Deformations, and Chromosomal Abnormalities (ICD-10 Q00-Q00)	Diseases of the Musculoskeletal System and Connective Tissue (ICD-10 M00-M99)	Diseases of the Skin and Subcutaneous Tissue (ICD-10 L00-L99)	Diseases of the Nervous System (ICD-10 G00-G99)	Diseases of the Digestive System (ICD-10 K00-K95)	Diseases of the Circulatory System (ICD-10 I00-I99)	Mental, Behavioral and Neurodevelopmental Disorders (ICD-10 F01-F99)	Diseases of the Genitourinary System (ICD-10 N00-N99)	Diseases of the Respiratory System (ICD-10 J00-J99)	Diseases of the Eye and Adnexa (ICD-10 H00-H59)	Diseases of the Blood and Blood-Forming Organs and Certain Disorders Involving the Immune Mechanism (ICD-10 D50-D89)	Endocrine, Nutritional and Metabolic Diseases (ICD-10 H00-H59)	Symptoms, Signs, and Abnormal Clinical and Laboratory Findings, Not Elsewhere Classified (ICD-10 R00-R99)	Diseases of the Ear and Mastoid Process (ICD-10 H60-H95)	Pregnancy, Childbirth, and the Puerperium (ICD-10 O00-09A)
Hearing loss: [14-30]	19 (90%)	15 (71%)	11 (52%)	5 (24%)	4 (19%)	4 (19%)	3 (14%)	2 (10%)	2 (10%)	1 (5%)	1 (5%)	1 (5%)	3 (14%)	0 (0%)	0 (0%)
Jaw Disorders: [19,21,31-36]	8 (100%)	6 (75%)	4 (50%)	4 (50%)	2 (25%)	1 (13%)	3 (38%)	0 (0%)	1 (13%)	3 (38%)	1 (13%)	2 (25%)	2 (25%)	2 (25%)	0 (0%)
Cleft Lip/Palate: [19,21,28,37-41]	8 (100%)	5 (63%)	3 (38%)	6 (73%)	2 (25%)	1 (13%)	3 (38%)	1 (13%)	0 (0%)	0 (0%)	1 (13%)	0 (0%)	2 (25%)	3 (38%)	0 (0%)
Thyroid: [42,43]	1 (50%)	1 (50%)	0 (0%)	2 (100%)	0 (0%)	0 (0%)	0 (0%)	0 (0%)	0 (0%)	0 (0%)	0 (0%)	1 (50%)	0 (0%)	0 (0%)	1 (50%)
Dysphagia: [19,44-47]	5 (100%)	4 (80%)	1 (20%)	4 (80%)	1 (20%)	1 (20%)	2 (40%)	0 (0%)	0 (0%)	1 (20%)	1 (20%)	0 (0%)	4 (80%)	0 (0%)	0 (0%)
Ear Deformity: [14,16,18,23,27,30]	6 (100%)	3 (50%)	2 (33%)	1 (17%)	0 (0%)	3 (50%)	0 (0%)	0 (0%)	0 (0%)	0 (0%)	0 (0%)	0 (0%)	1 (17%)	0 (0%)	0 (0%)
Overall Statistics**	40 (91%)	29 (66%)	18 (41%)	17 (39%)	6 (14%)	7 (16%)	6 (14%)	5 (11%)	2 (5%)	5 (11%)	1 (2%)	3 (7%)	8 (18%)	5 (11%)	1 (2%)

Next, the imaging and diagnostic tests were analyzed to understand the imaging needed for KFS patients (Table 9). It is important to note that many patients received more than one imaging study, accounting for more imaging studies included than patients.

**Table 9:** Diagnostic Imaging Ordered in Klippel-Feil Syndrome Patients CT: computed tomography, MRI: magnetic resonance imaging, US: ultrasound, ECG: electrocardiogram

Symptom	Imaging and Diagnostics: N (%)					
	CT	MRI	X-Ray	US	ECG	Others
Hearing loss: [14-30]	CT: 8 (38%)	8 (38%)	6 (29%)	4 (19%)	1 (5%)	2 (10%)
Jaw Disorders: [19,21,31-36]	4 (50%)	2 (25%)	3 (38%)	0 (0%)	0 (0%)	0 (0%)
Cleft Lip/Palate: [19,21,28,37-41]	5 (63%)	4 (50%)	3 (38%)	0 (0%)	0 (0%)	3 (38%)
Thyroid: [42,43]	0 (0%)	0 (0%)	1 (50%)	0 (0%)	0 (0%)	0 (0%)
Dysphagia: [19,44-47]	3 (60%)	4 (80%)	2 (40%)	0 (0%)	0 (0%)	0 (0%)
Ear Deformity: [14,16,18,23,27,30]	4 (67%)	3 (50%)	2 (33%)	3 (50%)	1 (17%)	0 (0%)
Overall Statistics**	24 (55%)	21 (48%)	17 (39%)	7 (16%)	3 (7%)	2 (5%)

Next, Mallampati score was analyzed. The patients who reported a Mallampati score are found in Table 10.

**Table 10:** Mallampati Scores in KFS Patients The classic triad includes a low posterior hairline, short neck, and limited cervical range of motion [2]. KFS: Klippel-Feil syndrome, ROM: range of motion, CT: computed tomography, US: ultrasound, MRI: magnetic resonance imaging, BLOOD: Diseases of the blood and blood-forming organs and certain disorders involving the immune mechanism, ENDO: Endocrine, nutritional and metabolic diseases, CNS: Mental, Behavioral and Neurodevelopmental disorders, NEURO: Diseases of the Nervous System, EYE: Diseases of the eye and adnexa, EAR: Diseases of the ear and mastoid process, CIR: Diseases of the Circulatory System, RESP: Diseases of the respiratory system, GI: Diseases of the digestive system, INT: Diseases of the skin and subcutaneous tissue, MSK: Diseases of the musculoskeletal system and connective tissue, GU: Diseases of the genitourinary system, PREG: Pregnancy, childbirth, and the puerperium, GEN: Congenital malformations, deformations, and chromosomal abnormalities, OTHER: Symptoms, signs, and abnormal clinical and laboratory findings, not elsewhere classified

Author	Age/Gender	Mallampati Score	Comorbidities and Characteristics	Cervical Fusion	Samartzis Classification	Imaging
Altay et al., 2014 [48]	26 y/o F	2	Intestinal obstruction (GI), heart failure (CIR)	Not listed	None Listed	ECG
Hase et al., 2014 [34]	27 y/o F	4	Limited cervical ROM (MSK), asymmetry of clavicle and scapula (GEN), limited arm ROM (MSK), deviated nasal septum (GEN), retrognathia (GEN)	C3-C5	Type 3	X-ray
Jena et al., 2022 [49]	10 y/o F	2	Short neck (GEN), scoliosis (GEN), platybasia (GEN), Chiari malformation (NEURO), systolic murmur (CIR), limited cervical ROM (MSK), b/l neck weakness (MSK), decreased limb sensation and weakness in all limbs (NEURO). Aortic stenosis (CIR)	C5-C7	Type 3	ECG, X-ray
Aravindan et al., 2021 [21]	38 y/o F	2	Short neck (GEN), webbed neck (GEN), limited cervical ROM (MSK), low posterior hairline (INT), ear discharge (EAR), sensorineural hearing loss (NEURO)	C1-C3	Type 3	Not Listed
Aravindan et al., 2021 [21]	18 y/o F	3	Unilateral hearing loss (NEURO), snoring (RESP), short neck (GEN), webbed neck (GEN), limited cervical ROM (MSK), low posterior hairline (INT), large tongue (GEN), bifid uvula (GEN), pectus carinatum (GEN), left thumb hypoplasia (GEN), absent metacarpal (GEN), intellectual disability (CNS), delayed sexual characteristics (ENDO), chronic otitis media (EAR), mayer rokitansky kuster hauser syndrome (GEN)	C2-C7	Type 3	Not Listed
Aravindan et al., 2021 [21]	21 y/o F	3	Amenorrhea (GU), short/webbed neck (GEN), reduced cervical ROM (MSK), low posterior hairline (INT), scoliosis (GEN), unilateral kidney agenesis (GEN)	C1-C2, C5-C6	Type 3	X-ray
Pacreu et al., 2018 [47]	43 y/o M	4	Nystagmus (EYE), facial hemianesthesia (GEN), dysphonia (OTHER), syringomyelia (NEURO), limited cervical ROM (MSK), low posterior hairline (INT), short neck (GEN), facial dysmorphism (GEN), Chiari disease (NEURO), limited jaw ROM	Cervical Vertebrae	Not Listed	MRI
Sirico et al., 2023 [29]	34 y/o F	3	Short neck (GEN), low posterior hairline (INT), limited cervical ROM (MSK), scoliosis (GEN), Sprengel deformity (GEN), b/l deafness (NEURO)	Atlanto-occipital joint, C2-C3	Type 1	MRI
Bakan et al., 2015 [50]	3 y/o F	3	Short neck (GEN), low posterior hairline (INT), Cervical lordosis (GEN), Sprengel’s deformity (GEN), encephalocele (NEURO)	C2-C3	Type 1	X-ray
Paramaswamy et al., 2019 [35]	16 y/o M	2	Limited cervical ROM (MSK), low posterior hairline (INT), misaligned teeth (GI), mandibular prognathism (GEN), overbite (GI), psychological disturbances (NEURO), kyphoscoliosis (GEN),	C1-C6	Type 3	X-ray CT
Zhang et al., 2021 [51]	8 y/o M	3	Torticollis (GEN)	Occipito-atlanto deformity	Not Listed	US
Xu et al., 2021 [40]	41 y/o F	4	Neurologic symptoms (NEURO), cervical spinal cord compression (GEN), severe canal stenosis and retrolisthesis, scoliosis of cervicothoracic spine (GEN)	C4-C5	Type 1	X-ray, MRI, CT
Munter et al., 2014 [41]	28 y/o F	3	Cleft Palate (GEN), abnormal face (GEN), sprengel deformity (GEN), scoliosis, (GEN) C3-C4 stenosis (GEN)	C2-C3	Type 1	ECG
Kavanaugh et al., 2013 [39]	38 y/o F	4	Cleft palate (GEN), chronic neck and back pain (MSK), cervical spina bifida cystica myelomeningocele (NEURO), multiple cleft vertebrae (GEN), kyphoscoliosis (GEN), limited mouth opening (MSK), short neck (GEN), limited cervical ROM (MSK), thoracic and lumbar scoliosis (GEN), Sprengel deformity (GEN), webbed neck (GEN), large foramen magnum (GEN),	C2-C4, C5-C7, T3-T5	Type 3	MRI

Next, the patients who reported a Cormack-Lehane grade were analyzed. These findings are found in Table 11.

**Table 11:** Cormack-Lehane Grade in KFS Patients The classic triad includes a low posterior hairline, short neck, and limited cervical range of motion [2]. KFS: Klippel-Feil syndrome, ROM: range of motion, CT: computed tomography, US: ultrasound, MRI: magnetic resonance imaging, BLOOD: Diseases of the blood and blood-forming organs and certain disorders involving the immune mechanism, ENDO: Endocrine, nutritional and metabolic diseases, CNS: Mental, Behavioral and Neurodevelopmental disorders, NEURO: Diseases of the Nervous System, EYE: Diseases of the eye and adnexa, EAR: Diseases of the ear and mastoid process, CIR: Diseases of the Circulatory System, RESP: Diseases of the respiratory system, GI: Diseases of the digestive system, INT: Diseases of the skin and subcutaneous tissue, MSK: Diseases of the musculoskeletal system and connective tissue, GU: Diseases of the genitourinary system, PREG: Pregnancy, childbirth, and the puerperium, GEN: Congenital malformations, deformations, and chromosomal abnormalities, OTHER: Symptoms, signs, and abnormal clinical and laboratory findings, not elsewhere classified

Author	Age/Gender	Cormack-Lehane Grade	Comorbidities and Characteristics	Cervical Fusion	Samartzis Classification	Imaging
Hase et al., 2014 [34]	27 y/o F	Grade 4	Limited cervical ROM (MSK), asymmetry of clavicle and scapula (GEN), limited arm ROM (MSK), deviated nasal septum (GEN), retrognathia (GEN)	C3-C5	Type 3	X-ray
Pagano et al., 2019 [46]	70 y/o M	Grade 2A	Scoliosis (GEN), prognathism (GEN), airway management difficulties (GEN)	Not listed	Not listed	CT
Dialameh et al., 2023 [52]	27 y/o M	Grade 4	Short neck (GEN), limited cervical ROM (MSK), scoliosis (GEN), ptosis of right eye (EYE), ectopic pelvic kidney on right side (GU), kidney failure (GU), dextrocardia (CIR), coarctation of the aorta (CIR), left ventricular hypertrophy (CIR), aortic insufficiency (CIR)	C3-C5	Type 3	ECG, CT, X-ray
Bakan et al., 2015 [50]	3 y/o F	Grade 1	Short neck (GEN), low posterior hairline (INT), Cervical lordosis (GEN), Sprenge’s deformity (GEN), encephalocele (NEURO)	C2-C3	Type 1	X-ray
Xu et al., 2021 [40]	41 y/o F	Grade 2A	Neurologic symptoms (NEURO), intellectual disability (NEURO), cleft palate (GEN), scoliosis (GEN)	C4-C5	Type 1	MRI, X-ray, CT

Finally, the Mallampati score and Cormack-Lehane grade were analyzed (Table 12). Patients were stratified by their respective grades and Samartzis classification. Three patients reported both the Mallampati score and Cormack-Lehane grades.

**Table 12:** Mallampati Score and Cormack-Lehane Grade Stratified by Samartzis Classification N: number of patients, SD: standard deviation

Test	Sample Size	Gender N (%)		Age (Years) Mean (SD)	Mallampati Score or Cormack-Lehane Grade by Number of Patients (%)			
		Male	Female		Class 1	Class 2	Class 3	Class 4
Mallampati Score: [21,29,34,35,39,40,41,47-51]	14 cases	4 (29%)	10 (71%)	25.1 (12.88)	0 (9%)	4 (28.5%)	6 (43%)	4 (28.5%)
Cormack-Lehane Grade: [34,40,46,50,52]	5 cases	2 (40%)	3 (60%)	33.6 (24.51)	1 (20%)	2 (40%)	0 (0%)	2 (40%)

**Figure 2:**
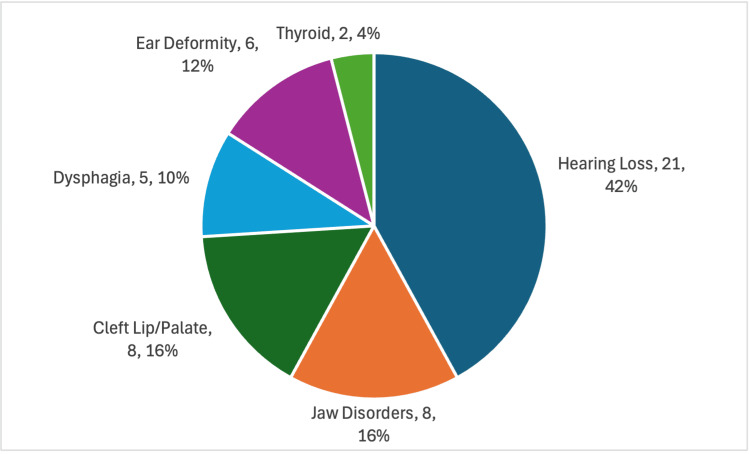
Proportion of Otolaryngological-Related Symptoms Data is presented as (N, %).

Results show that KFS with ENT-related symptoms occurs more in females (28, 64%) than males (16, 36%). Within KFS patients who reported ENT symptoms, Figure 2 shows the proportion of each symptom (hearing loss, jaw disorders, cleft lip/palate, dysphagia, ear deformity, and thyroid). The results show that hearing loss is the most common symptom associated with KFS (21, 42%) and there were the least amount of patients that presented with thyroid disorders (2, 4%). Several patients presented with multiple ENT-related symptoms explaining why the number of patients in Figure 2 adds up to more than the 44 included in this review. From cases that presented with ENT symptoms, 13 (35%) cases presented with more than one KFS symptom.

Samartzis Classification

Classification of cases using the Samartzis classification system demonstrated that patients with multiple, noncontiguous fusions in the cervical region (type III) were found to have more severe symptoms related to otolaryngology when compared to patients classified as type I [14-47]. More severe Smaratzis classification patients had more ENT-related issues and more severe issues like hearing loss, jaw disorders, cleft lip/palate, and dysphagia [14,16,17,19,21,28,31,39,45].

Hearing Loss

A total of 21 patients reported with hearing loss [14-30]. Of these, 12 (57%) were type 3 Samartzis fusions, five (24%) were type 1 Samartzis fusions, and four (19%) were unreported. These findings can be seen in Table 7. This supports that more severe fusions are correlated with more severe side effects. There were 13 (62%) females and eight (28%) males that presented with hearing loss. These patients presented at an average age of 23.35 years. Varying types of hearing loss were reported including sensorineural, conductive, and mixed. There were nine (43%) cases that reported sensorineural hearing loss [14-16,18,21,25,27,29], three (14%) that reported conductive [25,26], and three (14%) that reported mixed [19,30,23]. The remaining six (29%) cases did not specify the type of hearing loss [14,17,20,22,24,28]. The most common symptoms that occur with hearing loss in KFS patients are additional congenital malformations, deformations, and chromosomal abnormalities (19, 90%). This is followed by diseases of the musculoskeletal system (15, 71%) and diseases of the skin and subcutaneous tissue (11, 52%).

Jaw Disorders

A total of eight cases presented with jaw disorders, including retrognathia, micrognathia, and prognathism [19,21,31-36]. Of the cases reported, five (63%) were type 3 fusions, one (13%) were type 1 fusions, and one (13%) was not listed. Further, five (63%) were females, and three (38%) were males. The most common symptoms that occur with jaw disorders are additional congenital malformations, deformations, and chromosomal abnormalities (8, 100%), diseases of the musculoskeletal system (6, 75%), and diseases of the nervous system (4, 50%) and Diseases of the skin and subcutaneous tissue (4, 50%).

Cleft Lip/Palate

A total of eight cases presented with cleft lip/palate [19,21,28,37,38-41]. Of the cases reported, four (50%) were type 3 fusions, three (38%) were type 1 fusions, and one (13%) was not listed. Further, seven (88%) of the reported cases were female and one (13%) was male. The most common symptoms that accompany cleft lip/palate are additional congenital malformations, deformations and chromosomal abnormalities (8, 100%), followed by diseases of the nervous system (6, 73%) and diseases of the musculoskeletal system (5, 63%).

Dysphagia

Five cases reported dysphagia, with three (60%) being type 3 fusions, and two (40%) not listed [19,44-47]. Further, three (60%) cases were males, and two (40%) were females. The most common symptoms seen in KFS patients with dysphagia are additional congenital malformations, deformations and chromosomal abnormalities (5, 100%), followed by diseases of the nervous system (4, 80%) and diseases of the musculoskeletal system (4, 80%).

Ear Deformity

Six cases presented with ear deformities [14,16,18,23,27,30]. Of the cases reported, three (50%) were type 1 fusions, two (33%) were type 3 fusions, and one (17%) was not listed. Further, three (50%) were males, and three (50%) were females. The most common additional symptoms that presented in addition to ear deformity are additional congenital malformations, deformations and chromosomal abnormalities (6, 100%), followed by diseases of the musculoskeletal system (3, 50%) and diseases of the circulatory system (3, 50%).

Thyroid

Two cases reported thyroid symptoms [42,43]. Of the cases reported, two (100%) were female. Additionally, one (50%) was a type 1 fusion, and one (50%) was not listed. Finally, two (100%) cases reported severe hypothyroidism.

Imaging

Analysis of the 44 cases included in this review revealed a total of 74 orders for diagnostic imaging [14-47]. CT was the most common test ordered, with 24 (55%) scans. MRI was second most common with 21 (48%) scans, and X-ray was third most common with 17 (39%) scans. These scans were used and ordered to identify or confirm spinal cord fusions; however, patients often had additional abnormalities found during imaging. It should be noted that many patients received multiple scans.

Mallampati Score

The Mallampti scoring system defines patients with a score greater than 2 as having an abnormal airway [11]. Fourteen cases included in this review reported Mallampti scores [48,34,49,21,47,29,50,35,51,40,41,39]. Of the cases reported, four (29%) patients reported class 2, six (43%) patients reported class 3, and four (29%) patients reported class 4. Of cases reporting Mallampati scores, 10 (71%) patients presented with an abnormal Mallampati score.

Cormack-Lehane Grade

Five cases reported Cormack-Lehane grades, which also correlate with difficulties in airway management [12,34,46,52,50,40]. Of reported cases, one (20%) reported a grade 1 Cormack-Lehane grade, two (40%) reported grade 2, and two (40%) patients with grade 4 [12]. Similar to the Mallampati score, a higher grade is associated with an abnormal airway that can cause problems with intubation and breathing, and a score of 2 or lower is typically considered normal [12].

Discussion

Overview

This systematic review reveals that ENT-related symptoms are more prevalent and severe in patients with type 3 Samartzis fusions compared to those with type 1 fusions. Type 2 fusions are extremely rare, and no cases with ENT-related symptoms were found in this review. KFS is extremely rare and often presents with additional symptoms accompanying cervical fusions. Body systems most affected by spinal cord fusion were also identified. In addition to otolaryngological symptoms, patients with KFS frequently have cardiac, renal, and neurological abnormalities, necessitating a multidisciplinary treatment approach to provide comprehensive care.

Symptoms That Accompany Fusion

The analysis included specific ENT symptoms such as hearing loss, jaw disorders, cleft lip/palate, dysphagia, ear deformity, and thyroid malfunction, and examined what other symptoms and body system abnormalities KFS patients have. It was found that 91% of KFS patients present with additional congenital malformations, deformations, and chromosomal abnormalities, such as Sprengel’s deformity and scoliosis. Cardiac malformations are also common. Almost every patient had at least one additional congenital malformation alongside KFS, underscoring the importance of imaging to identify these conditions early, even if the patient is asymptomatic, to prevent future health issues and enhance the quality of care.

Beyond congenital malformations, KFS patients frequently have musculoskeletal diseases (66%), integumentary diseases (41%), and nervous system diseases (39%). Spina bifida, decreased cervical range of motion, and a low posterior hairline are common symptoms. These findings highlight the importance of considering these symptoms when evaluating patients for KFS.

KFS is classically described by a triad of a short neck, low posterior hairline, and limited cervical range of motion [53]. However, in patients with ENT symptoms, only 26% presented with the classic triad, indicating that most patients present with only one or two of these features. Therefore, the absence of the classic triad should not rule out a KFS diagnosis.

Hearing Loss Analysis

Hearing loss is a significant comorbidity in KFS patients and is more common in those with severe cervical fusions. Many KFS patients with hearing loss also have ear malformations such as malformed semicircular canals or tympanic membranes. Otolaryngologists must be aware of these potential malformations as they can influence treatment strategies.

Hearing loss in KFS can be sensorineural, conductive, or mixed. Sensorineural hearing loss is due to problems in the inner ear or along the auditory nerve pathways leading to the brain, conductive hearing loss occurs when there are problems with the outer or middle ear that prevent conduction of sound to the inner ear, and mixed hearing loss is a mix of both types of hearing loss [54]. While all types of hearing loss were found in KFS patients, sensorineural hearing loss was found to be the most common. This underscores the need for a comprehensive workup to understand the pathophysiology and ensure accurate diagnosis. It's also crucial to consider KFS in the differential diagnosis when evaluating patients with hearing loss and neck or spinal malformations.

Interestingly, hearing malformation did not follow the same gender or severity trends as other ENT symptoms. The distribution was equal between males and females, with a higher occurrence in type 1 fusions compared to type 3. However, these results may be influenced by the small sample size, and further research is needed to draw definitive conclusions.

Airway Analysis

Airway management is critical in KFS and was analyzed through Mallampati and Cormack-Lehane scores. Of the cases reported, 71% had an abnormal Mallampati score (Class 3 or 4), and 40% had a Cormack-Lehane grade 4 airway. As higher Mallampati scores correlate with an increased risk of a difficult intubation, an ENT consultation is recommended for scores greater than 2 as these procedures may necessitate special techniques [55]. Higher Mallampati scores are also associated with an increased risk for obstructive sleep apnea, which can significantly affect the quality of life for KFS patients. Given the frequent need for corrective surgery in KFS patients, these considerations are essential for improving surgical outcomes and quality of life.

Imaging

Imaging is another vital part of the KFS workup. The most commonly used imaging tests for KFS patients are CT scans, followed by MRI and X-rays. These imaging techniques enable visualization of the spinal cord fusions required for a KFS diagnosis. According to the findings in Table 3, patients should initially undergo an X-ray or CT scan for preliminary visualization, with an MRI reserved for further assessment based on initial results. This approach is due to the high costs of MRI and the fact that it may not be necessary for all patients. Additional imaging and lab tests should be customized to address the patient's specific symptoms.

Patient Education and Referral

Given the complexity of KFS, it is vital for the primary care provider (PCP) to be informed of the diagnosis. KFS patients often need a multidisciplinary team, including neurosurgery, orthopedic surgery, cardiology, and genetics specialists, to manage their symptoms effectively. Proper patient education is crucial because patients with certain types of fusions are at increased risk of serious spinal injuries from minor trauma. Several KFS patients have reported quadriplegia or complete locked-in syndrome following simple accidents [30,56-62]. Therefore, patients need to be counseled on their increased risks, and physicians must be aware to provide appropriate education and preventive advice.

Limitations

The primary limitation of this systematic review stems from the limited data on KFS. This is a rare occurrence seen in approximately 1 in 42,000 live births [1,7]. While the true prevalence may be higher due to asymptomatic and unreported cases, the small number of reported cases constrains the data and analysis. Additionally, variability in diagnostic criteria and potential biases in included studies may affect the generalizability of the findings. Further research with larger sample sizes is needed to validate these results and provide more robust conclusions.

## Conclusions

KFS, though rare, significantly impacts affected individuals with symptoms including neck pain, hearing loss, dysphagia, scoliosis, and other musculoskeletal anomalies. Otolaryngologists should include KFS in their differential diagnosis when encountering such symptoms. Early diagnosis, confirmed through imaging, is crucial, as KFS patients often face increased risks of paralysis, reduced mobility, and require frequent medical care. A multidisciplinary approach involving primary care, genetics, neurosurgery, and orthopedics is essential for managing the complex needs of KFS patients and improving their quality of life. Enhanced research and case reporting are needed to better understand and manage this syndrome. By remaining alert to the possibility of KFS and providing coordinated multidisciplinary care, healthcare professionals can greatly improve outcomes and quality of life for individuals affected by this complex syndrome.
