# Correction: Otolaryngological Presentations of Klippel-Feil Syndrome: A Systematic Review

**DOI:** 10.7759/cureus.c325

**Published:** 2025-09-26

**Authors:** Christopher Stewart, Alex L Otto, Mitchell Fisher, Abbigail Niewchas, Salma Alkhatib, Andrew Simonsen, Randall Hansen, Suporn Sukpraprut-Braaten, Kent McIntire

**Affiliations:** 1 College of Medicine, Kansas City University, Joplin, USA; 2 Otolaryngology - Head and Neck Surgery, Freeman Health System, Joplin, USA; 3 Graduate Medical Education, Kansas City University, Joplin, USA

This article has been corrected as follows:

- PRISMA diagram: "Studies included in review (n = 46)" corrected to "Reports included in review (n = 39), Patient cases described in 39 reports selected (n = 45)"
- Results section, first paragraph: "A total of 44 cases were analyzed." corrected to "A total of 44 cases with reported ENT abnormalities were analyzed. (There was one patient that reported a Mallampati score but had no abnormalities.)"

